# Comparative Analysis of Sandwich Composites with Balsa, Rohacell^®^, and Nomex^®^ Cores for Aerospace Applications

**DOI:** 10.3390/ma18051126

**Published:** 2025-03-02

**Authors:** Joanna Pach, Roman Wróblewski, Bartłomiej Muszyński

**Affiliations:** Department of Lightweight Elements Engineering, Foundry and Automation, Faculty of Mechanical Engineering, Wroclaw University of Science and Technology, Wyb. Wyspiańskiego 27, 50-370 Wrocław, Poland; bartlomiej.muszynski01@gmail.com

**Keywords:** sandwich composites, polymer-matrix composites, quasi-static penetration test, bending test

## Abstract

Interlayered composites with three types of cores were fabricated and tested. Quasi-static penetration tests (QSPTs), bending tests, and impact tests were conducted on the fabricated composites with carbon fiber epoxy laminate facings. Penetration test procedures were carried out until the composite was perforated and completely punctured. A 9 mm diameter rounded-tip punch was used; the diameter of the support hole was 45 mm. To determine the mechanical properties in the bending tests, three-point bending was carried out at a speed of 2 mm/min. Impact tests were also carried out using a Charpy impact test and a hammer with an energy of 2 J. Our findings indicate that the core material plays a crucial role in determining a composite’s mechanical behavior. Balsa cores offer the best properties in the QSPT test and bending strength and stiffness (57 MPa and 7.4 GPa, respectively), while Rohacell^®^ cores provide excellent impact resistance (12 kJ/m^2^). Nomex^®^ cores demonstrate high bending stiffness (5.3 GPa) but perform worse than Balsa. The choice of core material is application-dependent; Balsa cores are optimal for bending and point loads, and Rohacell^®^ cores are optimal for impact-dominated scenarios.

## 1. Introduction

Structural materials in aviation have evolved significantly over the years, from the wooden frame of the Wright brothers’ Flyer I to the metal Junkers J-1 and the composite Airbus A350. The demand for lighter and stronger materials has grown alongside increasing aircraft performance. The Wright Flyer was the first aircraft to demonstrate the feasibility of powered flight. Although the metal construction of the Junkers J-1 was not a successful design, it laid the groundwork for future metal-based aircraft. The shift to metal construction enabled the development of General Aviation aircraft, which were designed in the post-war period. These aircraft continue to be produced today and serve their purpose well.

Composite materials were initially introduced in military aircraft. From 1978, one of the first applications of modern composite materials was a boron-fiber-reinforced epoxy composite used for the fuselage skins of American F14 and F15 fighters. Initially, composite materials were primarily used in secondary structures [1].

Passenger aircraft began to adopt innovations introduced in military aviation, including epoxy-based composites. The primary benefit of using composite materials was reduced aircraft weight, leading to improved fuel efficiency, lower operating costs, and reduced CO_2_ emissions. Airbus quickly recognized these advantages and, in the 1980s, began implementing composite materials in the control surfaces of its commercial models, such as the A300 and A310 [2].

Today, the most advanced aircraft models use carbon fiber-reinforced composites. In the B787 model, composites account for approximately 50% of the structural mass [3,4]. The wide-body Airbus A350 XWB, constructed with over 54% composites (along with the latest Rolls-Royce Trent XWB engines), achieved a 25% reduction in fuel consumption compared to competing aluminum aircraft (A330, B777) [5].

The production of lightweight structures with good strength and low weight is particularly desirable in aviation, where low weight is crucial for aircraft performance. A solution considered in modern designs is layered composites, which allow for the combination of several components—fiber reinforcement, polymer resin, and core—to create a material with desired properties. Combining the lightness and strength of traditional polymer composites with the selected properties of the intermediate layer, layered composites become an interesting structural material for modern applications. These composites consist of external laminate layers separated by a stiffening, three-dimensional core, which is usually a low-mass core [6]. The outer layers are most often laminates consisting of a polymer matrix and reinforcement in the form of glass, carbon, aramid, or basalt fibers [7,8]. The stiffening core structures can be made of metal [9] or polymer and are honeycomb structures, such as Nomex^®^, proprietary structures [10,11], or hybrid structures combining two types of cores [12]. Natural materials such as Balsa wood [8,13] and foam structures such as Rohacell^®^ [14,15] are also used as core materials.

The ability to choose three materials, from which the composite is most often composed, makes it possible to obtain a new, unique material. Through additional properties resulting from the incorporation of the core between the two load-bearing laminate layers, the composite thus produced acquires properties that are not possible with standard laminates or other groups of structural materials. Most often, the outer layers and their orientation are identical, but the choice of two different surface materials is allowed. This may be due to the need to use an outer layer for esthetic or protective reasons, and such a composite is then asymmetrical in the central plane.

The dynamic development of layered composites brings new types of materials and opens up possibilities for creating structures that were previously impossible. The greatest advantage of sandwich composites is their relative simplicity of construction, which gives designers greater freedom to create more optimized structures. Lighter structures allow for increased range and payload of the aircraft, as well as reduced emissions per unit of power, which is important in the context of current efforts to protect the environment [16].

The requirements for stiffening core materials are determined by the needs of the technologist and the designed structure. Strength properties include compressive strength, shear strength, Young’s modulus, and Kirchhoff’s modulus [6,17]. Composites have various properties that depend mainly on the production method, quality, and the type of base material, as well as the type and proportion of reinforcement of the covering laminate. The ability to adapt their properties to specific requirements is a great advantage. Composite structures stand out from traditional single-phase materials due to their unique properties, such as increased strength, improved wear resistance, sliding properties, and high corrosion resistance.

When designing a layered material, it is worth remembering that the combination of the top laminate with the core is associated with three problems related to the influence of temperature on the material: thermal expansion, degradation of elastic properties, and increased creep [18]. Thermal expansion refers to the change in material dimensions in response to a change in temperature. In the case of sandwich composites, the top laminate and the core may have different coefficients of thermal expansion, which can lead to thermal stresses and damage when these materials are joined and exposed to the environment [19]. Degradation of elastic properties refers to the loss of the material’s ability to return to its original shape after the removal of the load. High temperatures can weaken these properties in composites, which can affect their strength, especially in situations where they are subjected to loads. Increased creep is a phenomenon in which the material slowly deforms under a constant load over time, especially at higher temperatures. In the context of layered composites, this can lead to problems with structural integrity, as different layers of the material may creep at different rates. All of these aspects are important in the design and selection of materials for sandwich composites, especially in aviation applications, where materials are regularly exposed to various factors and temperature differences.

Due to the possibility of wide application, the structure of sandwich composites is widely tested for mechanical properties, especially in bending tests [20] and impact resistance tests [21,22], not only typical tests used for polymers and polymer composites, but also some advanced tests using a Hexapod testing device [23].

This paper presents a comparative analysis of sandwich composites with three different cores: Balsa, Rohacell, and Nomex. The choice of these three materials for research in this work is due to their popularity and frequent use in the aviation industry. Balsa was used as a core material between plywood layers in the British multi-role military aircraft de Havilland Mosquito, used during World War II. The current use of Balsa in composite structures is increasing due to its high strength-to-weight ratio, high stiffness, and origin from natural resources, which is an important aspect of current research.

Each of these materials represents different approaches to core construction, from natural to synthetic, offering unique combinations of properties that are key in various aviation applications. Studying these materials allows for a better understanding of their potential advantages and limitations. The selection of Balsa [13,24], Rohacell [25,26], and Nomex [27] as core materials for testing is based on their widespread use in the industry and their distinct characteristics, which allow for a comprehensive comparison of structural performance. These materials represent different generations of core materials in aerospace and composite applications, each with unique advantages and trade-offs. By selecting these three materials, the study captures the progression of core material development in the industry and allows for a direct comparison of their mechanical behavior. The results provide insights into their advantages and limitations, helping to make informed decisions when selecting core materials for specific applications. Ilavarasu et al. [27] compared sandwich composites with a Nomex and Rohacell core for aviation applications. The bird impact analysis carried out on a sandwich flat panel was discussed. These composites were also compared as materials for radome for airborne applications. A Nomex core sandwich composite was considered the better material for these applications because it met both the electrical and structural requirements. In the research work of Castro [28], impact test composites with Rohacell and Cork cores were compared. It was found that compared to high-performance foams, composites with a cork core have better energy absorption capacity with minimal damage. In a research study [29], the tests of the sandwich beams with Rohacell foam core and carbon fiber skin sheets in three point bending tests were carried out, and mechanical response and damage evolution were analyzed. Experimental tests and numerical simulations were performed using maximum principal stress criteria to predict the damage propagation in the core. The results showed two dominant damage mechanisms: core shear in short-span specimens and cladding wrinkling in longer-span specimens. Observations recorded with a high-speed camera revealed the dynamic nature of crack propagation in the core and debonding of the cladding due to excessive wrinkling. In [30] the mechanical and fatigue properties of sandwich panels with carbon fiber-reinforced polymer (CFRP) skins and balsa cores were analyzed. The composites were tested in three-point bending and Charpy impact tests. The main failure mechanisms in the bending test included cracking of the upper skin and crack propagation through the core to the lower skin, causing its debonding. It was shown that sandwich structures with balsa cores have good impact resistance, but their fatigue strength limits their use in dynamic conditions [30]. In Galos’s research, the mechanical properties of composite sandwich structures with balsa wood core and GFRP laminate facings were analyzed [13]. The main studies concerned the bending properties and the influence of the balsa wood structure on the mechanical strength. The samples consisted of balsa cores of different densities and GFRP laminate facings. In three-point bending tests it was observed that the bending strength was strongly dependent on the balsa density, with higher-density samples showing greater resistance to damage. The results showed that the dominant failure mechanisms were the core wall and wrinkling of the facings at higher loads.

Although the selected cores were previously described separately [24,31], the authors of the paper undertook a comparison of composites with the mentioned cores in order to find the optimal material solution that will be used to produce a structural element of an aircraft. In order to compare the composites, the cores were selected to have the same thickness. The facing laminates were made of carbon fibers and epoxy resin. Due to the aforementioned problem of local loads in this type of composite, the composites were subjected to quasi-static puncture tests (QSPTs), impact tests, and bending tests.

## 2. Experimental

### 2.1. Materials

In this work, an epoxy resin matrix was used, known for its high compatibility with core materials and fabrics, ensuring strong adhesion and minimal chemical reactions. The resins most commonly used in aerospace for wet hand lamination are epoxy resins of the LH group, characterized by low viscosity, good adhesion, and specific curing mechanisms that do not require elevated temperatures. This significantly facilitates the lamination and curing process, preventing thermal degradation and the warping of sensitive core materials and facing laminates. The low level of heat generation ensures a high level of reliability and safety of the finished composite, crucial for aerospace applications. These resins also provide resistance to environmental factors such as moisture, UV radiation, and temperature [32]. HAVEL’s LH-289 resin and H289 hardener were selected(Havel Composites, Poland, Cieszyn). A key feature of this resin is its low viscosity of 500–900 mPa·s at 25 °C, which promotes effective wetting of the fabric and easier elimination of air bubbles during lamination. The resin/hardener ratio is 100:33 as recommended by the manufacturer. The working time of the H289 resin/hardener system is 30 min, the time within which the mixture can be successfully processed before it starts to gel [33]. The physical properties of LH-289 resin are shown in Table 1.

A 200 g/m^2^ 3K carbon fabric was chosen as the cladding for the sandwich composites. This choice was based on the superior mechanical properties of carbon fabric, including high specific strength, stiffness-to-weight ratio, and fatigue resistance. Despite the higher cost compared to aramid and glass fabrics, carbon fabric offers significant performance advantages. After analyzing the cost and properties of available materials, carbon fiber was selected as the most suitable option, considering the current trends in aerospace and the crucial need for weight reduction in the rapidly evolving industry.

Balsa (Latin: *Ochroma pyramidale*) is a natural core material renowned for its outstanding mechanical properties, rivaling many synthetic core materials. It is one of the lightest wood species in the world, with an average density of 110 kg/m^3^ (Table 2). Balsa wood has been successfully used in various applications, including miniature aircraft models and small boats (rafts) [34].

The density of balsa is most dependent on the climate in which it grows; more than 95% of balsa comes from Ecuador [35], where it grows in tropical forests, contributes to its rapid growth and is about 30 m in 15 years. Despite being a hardwood, balsa is very soft and lightweight. Using balsa as a core takes advantage of the anisotropic properties of the wood, and by orienting the balsa grain vertically, the best compressive strength can be achieved. Composites with a Balsa core are not used as critical elements. Balsa, being a natural material, is susceptible to growth conditions, which means that cores extracted at different times may have slightly different mechanical properties. The Balsa core exhibits good compressive and tensile strength, high flexural stiffness, and good shear strength. These mechanical properties indicate its suitability for applications requiring high strength and minimal deformation. Its renewability, durability, compatibility with all common resins, excellent strength-to-weight ratio, operating temperature of up to 160 °C, and good mechanical bonding to the resin make it an attractive choice for the design of sandwich structures. The Balsa, Rohacell^®^, and Nomex^®^ data used in the work are shown in Table 3.

Rohacell^®^ 71 IG-F is a high-performance PMI (polymethacrylimide) foam produced by Evonik Foams, Inc., (Essen, North Rhine-Westphalia, Germany) with a closed-cell structure, resulting in low resin absorption on the core surface. This low resin absorption improves bonding strength, reduces weight, and enhances dimensional stability. The isotropic nature of the core provides consistent material characteristics, making it suitable for critical applications such as honeycomb structures in aircraft interiors and leading edges of control surfaces. Rohacell^®^ foams are typically used as cores in sandwich structures and are not intended for use as standalone structural elements due to their low shear strength and compressive strength in the out-of-plane direction. Rohacell^®^ foams are produced using a copolymer of methacrylonitrile (C_4_H_5_N) and methacrylic acid (C_4_H_6_O_2_), with the addition of several ingredients, including alcohol as a foaming agent. During the foaming process, the liquid copolymer solidifies. Subsequently, Rohacell^®^ is formed through a temperature treatment process in an oven, involving polymerization and curing [38]. The density of the core used is 75 kg/m^3^, and the maximum operating temperature is 180 °C. The microstructure of Rohacell^®^ foam is characterized by a closed-cell structure with a cell size of 400 μm.

The third core used in the work is ‘Nomex^®^ Aramid Paper Honeycomb’, a hexagonal-shaped cellular structure mimicking the shape of a honeycomb. The core material is made of aramid paper and produced by DuPont de Nemours, Inc. Nomex^®^ honeycomb (Easy Composites, Stoke-on-Trent, UK) is typically used as a core material in sandwich structures and is not intended for use as a standalone structural element in aircraft due to its low compressive strength and shear strength in the out-of-plane direction. Nomex^®^ honeycomb offers several advantages, including high specific strength, excellent fire resistance, and good dimensional stability. Nomex^®^ is the lightest core material used, with a density of 48 kg/m^3^, a cell size of 4.8 mm, and a maximum operating temperature of 180 °C.

Balsa (Easy Composites, Stoke-on-Trent, UK) exhibits higher compressive, tensile, and shear strengths compared to Nomex^®^ and Rohacell^®^. It is a more rigid and robust material, suitable for applications requiring high strength and stiffness. However, the variability of its properties, influenced by environmental conditions during tree growth (e.g., soil conditions, rainfall, and sunlight exposure), can lead to some uncertainty in its performance. Rohacell^®^ has lower compressive and tensile strengths than Balsa, but offers significant advantages such as consistent properties, good impact resistance, and excellent dimensional stability. This makes it suitable for applications where weight reduction is a priority, and moderate strength is sufficient. Nomex^®^ honeycomb, while having low strength as a standalone material, is suitable as a core in applications where low weight and moderate strength and stiffness are required. However, its strength values vary significantly depending on the direction of the applied load (in-plane vs. out-of-plane).

In summary, Balsa offers the highest strength but with some uncertainty due to material variability. Nomex^®^ provides a good compromise between lightness and strength, while Rohacell^®^ excels in weight reduction and consistent performance.

The main objective of this study is to investigate the behavior of sandwich composites with three different core materials (Balsa, Rohacell^®^, and Nomex^®^) by comparing their performance in quasi-static penetration, bending, and impact tests. This study aims to provide a comprehensive understanding of the behavior of these composites, contributing to their wider application in aerospace structures.

### 2.2. Samples Preparation

Samples with a solid core were prepared using the vacuum bag method, which ensures optimal bonding between the core and the cover layers. For samples with a cellular core whose cells are open, a wet hand lamination method was used to ensure proper resin infiltration into the core and prevent void formation.

To ensure consistent comparisons, all samples were fabricated with the same core thickness (10 mm), carbon laminates with a [0°/90°]2s plyconfiguration, and LH 289 epoxy resin with LH-289 hardener.

**Figure 1:**
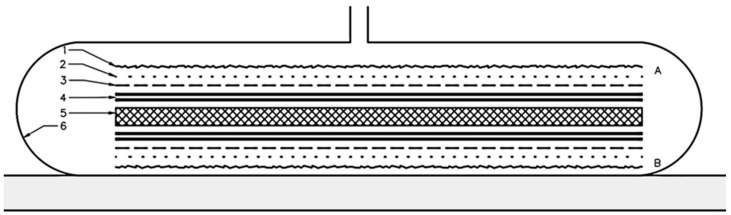
Layer configuration: 1—absorbent layer, 2—separation layer, 3—delamination, 4—two layers of carbon fabric, 5—core, and 6—vacuum bag. A—sample top, B—bottom of the sample.

Sandwich composites with solid cores were manufactured exclusively using the vacuum bag method (Figure 1). This process involved the use of auxiliary materials such as release film, breather cloth, and vacuum bagging film. The solid core was placed between the laminate layers, and the entire assembly was then placed in a vacuum bag. The vacuum provided uniform pressure, which helped to eliminate excess resin and air, ensuring optimal adhesion between the layers.

The process of producing samples with a cellular core, such as Nomex^®^, differs from the method used with solid cores, mainly due to its open cell structure. In this case, the vacuum bag method is not used. This is because excess resin used to bond the covers to the core could run down the walls of the core and accumulate on the bottom cover. This situation would lead to a poor bond between the top laminate and the core, which would reduce the quality of the overall composite. A very important element was the introduction of a delamination layer to prepare the surface of the laminate for bonding the cell core to it with resin. A resin was spread on the rough layer left by the delamination, which in this case, served as an adhesive to fix the core.

**Figure 2:**
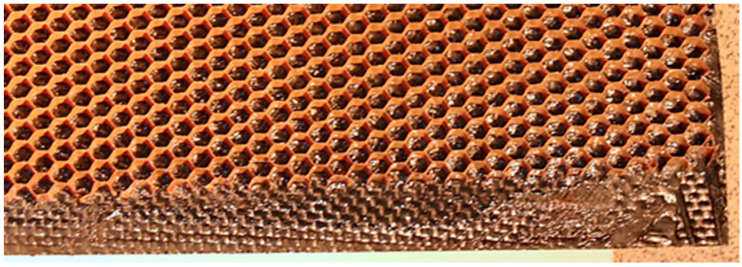
Cell core during the second stage of manufacturing.

The process of creating a cell-core sandwich composite can be divided into four main stages:

- 1: Two outer covers were prepared using a manual wet lamination process.
- 2: In total, 80 g of resin was applied to the carbon laminate surface, with the surface previously prepared, to adhere to the cell core. This configuration was left for 24 h to allow the resin to bond the core to the laminate layer. The thin layer of resin that bonds the core to the epoxy-carbon laminate is marked in Figure 2.
- 3: The same method as in step two was used to join the core set from the first cover to the second laminate.
- 4: After waiting 24 h, the spacer composite was ready to be cut.

**Figure 3:**
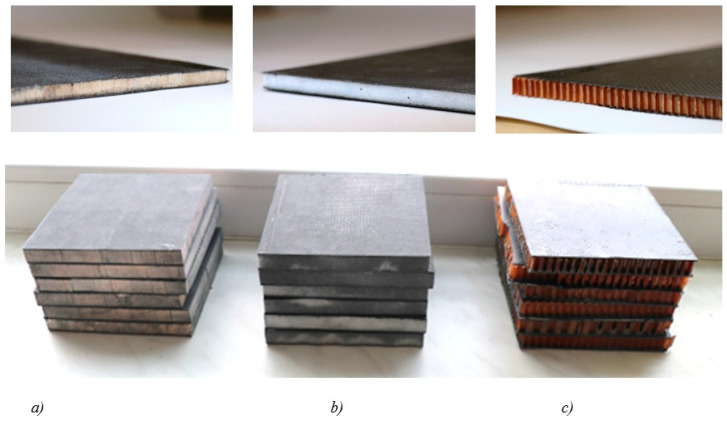
Cut samples with core: (**a**) Balsa, (**b**) Rohacell^®^, and (**c**) Nomex^®^.

After the composite structures were manufactured, their sizes were trimmed to 100 × 100 mm for the QSPT test. Specimens of 200 × 35 and 80 × 10 were prepared for the bending and impact test, respectively. Figure 3 shows cut and machined specimens with a dimension of 100 × 100 × 12 mm.

### 2.3. Methods

Three destructive tests were carried out as part of the test methods: quasi-static puncture test, bending test, and Charpy impact test. Each of these tests provided information on the mechanical properties of the fabricated structures. The experimental conditions were room temperature ±2 °C, and humidity 50 ± 10%.

#### 2.3.1. Quasi-Static Puncture Test (QSPT)

**Figure 4:**
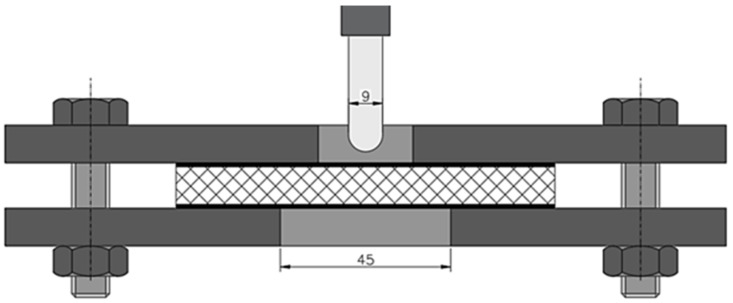
Schematic of specimen clamping during a quasi-static puncture test.

The puncture test was carried out using a Tinius–Olsen testing machine, in accordance with ASTM D732 [32]. A metal frame holding the specimen with a support bore diameter of 45 mm was used for the test (Figure 4). The intender had a diameter of 9 mm and a spherical shape. The ratio of the diameter of the punching pin to the diameter of the SPR support was equal to 5.0. The testing machine starts collecting data for the graph from the moment the punch pin makes contact with the surface of the specimen and ends when a displacement of 20 mm is reached. The machine beam moved at a speed of 1.25 mm/s. The test involves a punch test and is designed to determine the shear strength of organic plastic specimens in sheet form from 1.27 to 12.7 mm thick. The thickness of the test specimens is 12 mm and falls within the range quoted in the standard. The results that the quasi-static puncture test produces are the maximum force required to puncture, the puncture strength, the energy absorbed, and the specific energy.

Based on the data collected (averaged over five samples for each core), the puncture strength factor, the energy absorbed by the material, and the specific energy were calculated. Calculations were made based on Equations (1)–(4) [39].

The energy absorbed by the sample in a puncture test refers to the total amount of energy that the sample is able to absorb during the puncture process. The energy is represented by the box below the graph, which shows the relationship between the force applied and the displacement (position) of the piercing pin. The total absorbed energy (E_A_) was determined using the MatLab L2017b suite, using the trapezium integration method.

(1) $$E_{A}=\int_{0}^{20}f\left(x\right)dx$$

where integral limits are in the 0–20 mm range of the piercing pin displacement; f(x) is a function describing the dependence of the force on the displacement (position) of the stem.

SPR ratio—this is the ratio of the diameter of the punching pin to the diameter of the support hole.

(2) $$SPR=\frac{D}{\delta }=\frac{45\;\mathrm{mm}}{9\;\mathrm{mm}}=5\;$$

where

D—diameter of hole in plate, mm
δ—punch diameter, mm.

PSS—Punch Shear Strength (PSS) is an indicator used to assess a material’s ability to resist punching forces.

(3) $$PSS=\frac{F_{max}}{\pi \cdot \delta \cdot H_{c}}\;$$

where

F_max_—maximum punching force, N
δ—punch diameter, mm
H_c_—sample thickness, mm

SEA—(Specific Energy Absorption) is a measure of the amount of energy absorbed per unit mass of material.

(4) $$SEA=\frac{E_{A}}{m}\;$$

where

E_A_—energy absorbed by the sample
m—sample mass, kg.

#### 2.3.2. Three-Point Bending Test

**Figure 5:**
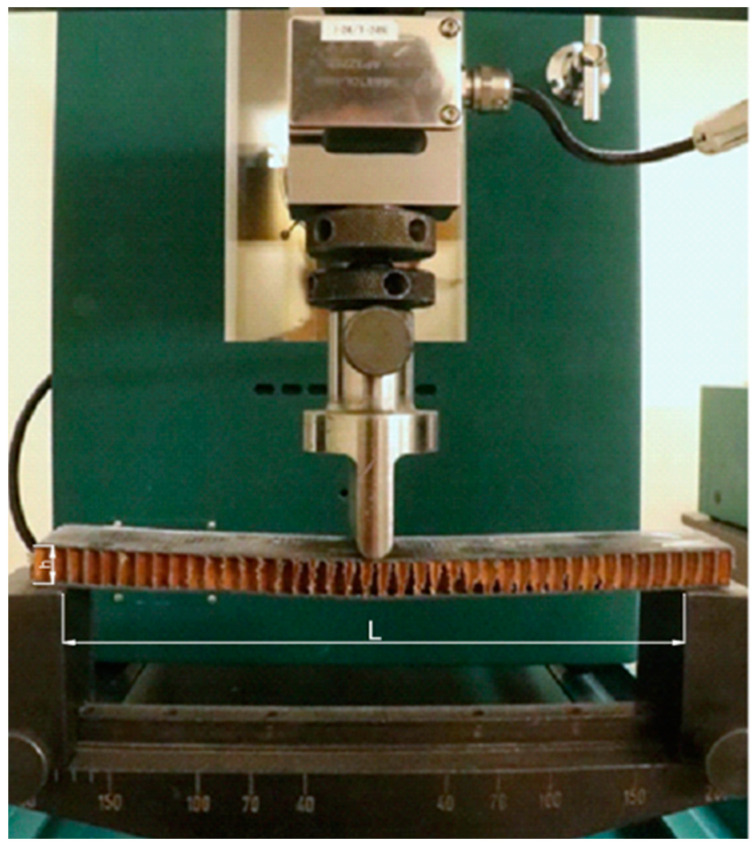
Three-point bending diagram, where R—bending mandrel radius, h—specimen thickness, l—specimen length, and L—support spacing width.

The three-point bending test was carried out on a Tinius-Olsen HKT25 testing machine (United Kingdom, Salfords). The test was mainly conducted for comparative purposes due to the numerous variables that can affect the results and the lack of an available standard covering sandwich composites. The test is based on ISO 178 [40]. During the test, the specimens are bent through a steel half-roll mandrel, placed at the top of the apparatus (Figure 5). The composites are subjected to bending at a crosshead displacement rate of 2 mm/min, and the main aim of the test is to observe the critical moment of loss of load bearing capacity of the composites and to measure the relationship between bending force and mandrel displacement. Figure 5 shows a diagram of three-point bending. The support spacing was 192 mm. Specimens of 200 × 35 × 12 mm were prepared. The flexural stress at break was calculated according to Formula (5).

- Flexural stress at break (σ_fB_):

(5) $$\sigma_{fB}=\frac{3F_{B}L}{2bh^{2}}\;$$

where

$F_{B}$—force at specimen failure, N
b—sample width, mm
h—sample height, mm
L—support spacing, mm.

#### 2.3.3. Charpy Impact Test

The Charpy impact test is a procedure that involves dynamically bending a rectangular specimen using a Charpy hammer, placed on two supports. The test was carried out partly on the basis of EN ISO 179 [41]. The specimens were impacted from two sides: from the laminate side and from the core side. For the impact test, the calculation of the impact factor is foreseen, which is determined as follows:

- Impact factor (KC):

(6) $$KC=\frac{K}{b\cdot h}$$

where

K—impact energy, J
b—sample width, mm
h—sample height, mm.

## 3. Results and Discussion

### 3.1. Quasi-Static Puncture Test

The experimental procedure of progressive failure was carried out on sandwich composites under quasi-static conditions.

**Figure 6:**
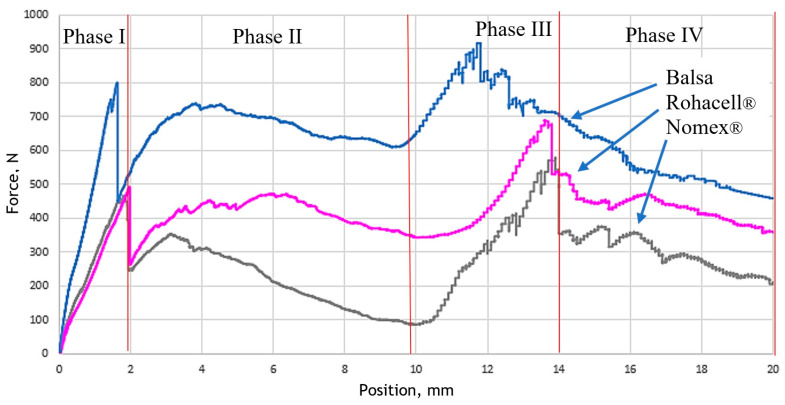
Comparison of quasi-static puncture test results.

Quasistatic-puncture tests were conducted for laminates with carbon fibers, both on matrix such as epoxy resin [42], polyurethane–polyurea resin [43], and thermoplastic [44]. The mechanism of damage to such laminates is well described in the literature [45] and includes three stages: elastic, damage, and friction. In the first stage, the penetration force increases linearly and elastic deformations arise in the material. In the damage stage, the fibers are destroyed and the matrix is damaged, followed by the formation of a plug [43,45]. The last stage of sample puncture is friction. In the case of sandwich composites, two peaks are observed on the F/displacement curve, which are related to the puncture of the laminate facing, as shown in Figure 6.

Figure 6 shows the force-displacement relationships for the interleaved composites tested, which most closely approximate the mean values.

In the first phase of the experiment, the sample with the Balsa core exhibited a rapid initial force increase, reaching a value of approximately 800 N. This was primarily attributed to the higher Young’s modulus of Balsa compared to the other cores. In the case of the Rohacell^®^ and Nomex^®^ core samples, an increase in force was also observed, with the maximum force being lower than that of the Balsa core, reaching a value of approximately 450 N. The breakthrough of the top cover in each specimen occurred abruptly, leading to a sudden drop in the force measured by the testing machine. This phenomenon was observed for all core types.

In the second phase, during the passage of the punch pin through the core, Nomex^®^ showed the greatest decrease in force. This was likely due to the relatively large cell size of the Nomex^®^ honeycomb compared to the diameter of the punch pin, which did not provide significant resistance to the penetrating punch. In contrast, the Rohacell^®^ and Balsa cores exhibited similar transition characteristics, with Balsa requiring the highest force for further punching. The Rohacell^®^ core, characterized by a fine cellular porous structure, underwent elastic and then plastic deformation as the punch pin penetrated. In the case of the Balsa core, which is more compact, the piercing pin compressed the material, leading to plastic deformation and subsequent shearing.

In the third phase, for Rohacell^®^ and Nomex^®^, the maximum force occurred just before the rapid puncture of the lower laminate layer (when the punch pin was at a depth of approximately 13 mm). For Balsa, the maximum force occurred earlier due to material accumulation and sliding along the punch pin, resulting in a less abrupt core penetration.

The fourth phase represents the passage of the punch pin through the entire sandwich composite, characterized by friction between the punch pin and the core and cover laminates. The force recorded by the testing machine decreased at a similar rate.

The sandwich composite with a Balsa core exhibited the best overall mechanical properties among the samples tested in the quasi-static puncture test.

**Figure 7:**
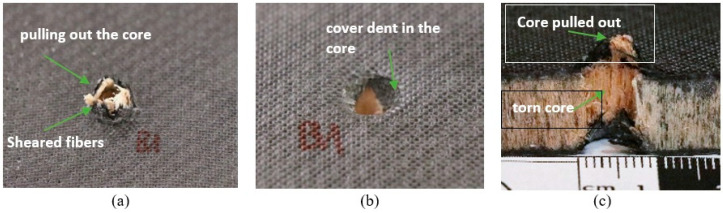
Example specimen after puncture for Balsa core; (**a**) Rear, (**b**) Front, (**c**) Cross-section.

**Figure 8:**
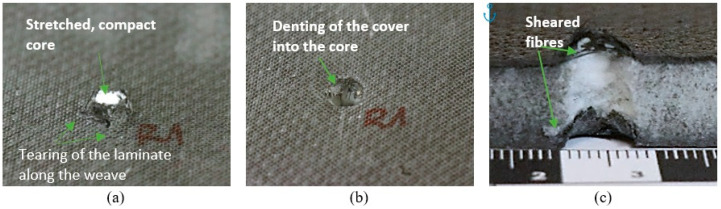
Example specimen after puncture for Rohacell^®^ core; (**a**) Rear, (**b**) Front, (**c**) Cross-section.

**Figure 9:**
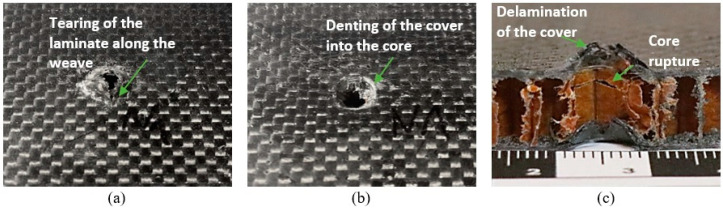
Example specimen after puncture for Nomex^®^ core: (**a**) rear, (**b**) front, and (**c**) cross-section.

Figure 7a,b, Figure 8a,b and Figure 9a,b characterize the sandwich composites, showing the front and back sides of the example specimens in close-up and providing a visual analysis of the damage. During the QSPT test, the increase in punch load up to the long tail friction stage resulted in damage mechanisms consisting of fiber fracture, cracking of the cover laminate, and subsequent cork formation, followed by the shearing of the cork from the specimens and cracking of the subsequent cover laminate.

Visual analysis revealed similar penetration marks on the entry side of all three composite types, characterized by specific features, e.g., circular shape and minimal material displacement. After the penetration tests, the specimens were cut along half of the damaged area with a saw to better understand and assess the damage during the QSPT test, as shown in Figure 7c, Figure 8c and Figure 9c. In the central part, i.e., in the core areas, some differences were observed after the passage of the penetrating punch pin due to the structure of the core itself. In the case of the Balsa core, the area under punch action had compressed and formed a plug pushed by the punch. Figure 7c shows the behavior of the area around the punch action, with the material partly sheared and partly pulled and pushed outward. In the case of the Nomex^®^ core (Figure 8c), the penetrating punch pin deformed the core slightly, mainly in the penetration region. In the case of the Rohacell^®^ core (Figure 9c), the jagged appearance of the core structure after penetration may indicate that the high plastic deformation of the core material induced significant tensile stresses in the boundary region, leading to irregular core separation.

**Figure 10:**
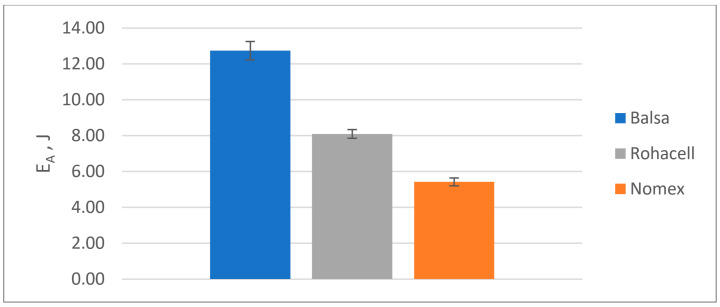
Energy absorbed (E_A_) in a quasi-static puncture test by sandwich composites with a different core.

**Figure 11:**
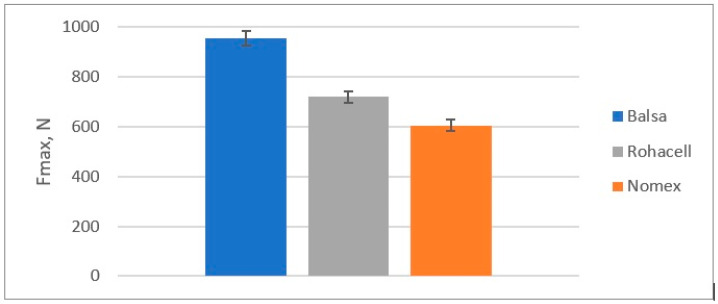
Maximum punching force (F_max_) in a quasi-static puncture test by sandwich composites with a different core.

**Figure 12:**
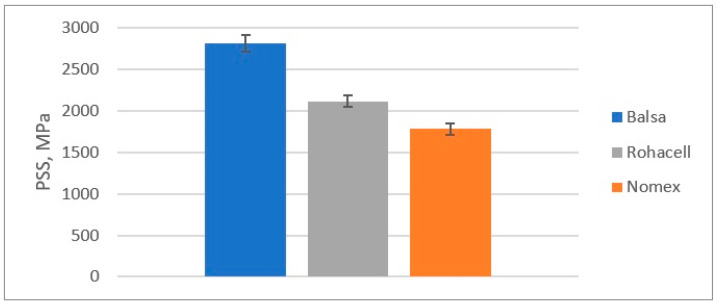
Punch Shear Strength (PSS) in a quasi-static puncture test by sandwich composites with a different core.

**Figure 13:**
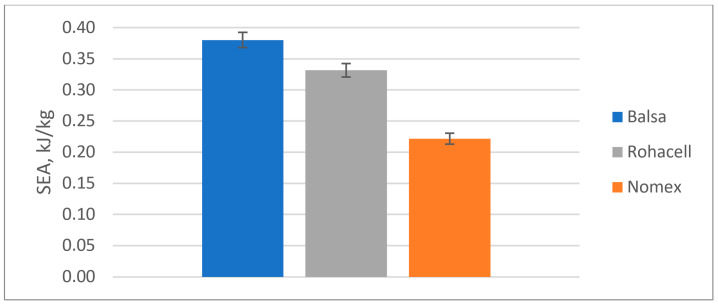
Specific Energy Absorbed (SEA) in a quasi-static puncture test by sandwich composites with a different core.

The results of the quasi-static puncture test calculations are shown in Table 4 and on Figure 10, Figure 11, Figure 12 and Figure 13.

- Energy absorbed (E_A_):

- Maximum punching force of the sample (F_max_):

- The Punch Shear Strength (PSS) of the tested samples:

- Specific Energy Absorbed (SEA):

Comparison of the results of the quasi-static punch test:

- Balsa shows the highest energy absorption capacity (SEA), which is 33% higher than Rohacell^®^ and 58% higher than Nomex^®^, suggesting that it is the best at absorbing impact or punching forces;
- Maximum force to puncture the specimen (Fmax)-Balsa also shows the highest value, which is 33% higher than Rohacell^®^ and 58% higher than Nomex^®^, meaning it can withstand the highest load before punching;
- Balsa has the highest puncture resistance, which is 57% higher than Rohacell^®^ and 135% higher than Nomex^®^, indicating its highest puncture resistance compared to Nomex^®^ and Rohacell^®^;
- Balsa offers the best energy absorption (SEA) per unit mass, 15% higher than Rohacell^®^ and 71% higher than Nomex^®^.

### 3.2. Determination of Impact Strength

**Figure 14:**
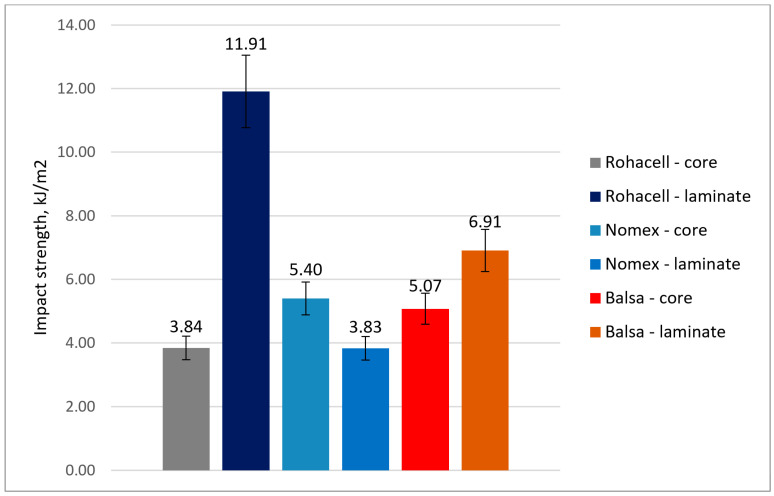
Charpy impact strength of tested composites.

The results of the Charpy impact test calculations are shown on Figure 14. By analyzing the values of the impact coefficient (IC), it can be observed that:

- The Balsa core composite has an impact strength of 5.07 kJ/m^2^ when impacted from the core side and 6.91 kJ/m^2^ when impacted from the laminate side.
- Rohacell^®^ core composite impacted from the laminate side significantly higher impact strength (11.91 kJ/m^2^) compared to impact from the core side (3.84 kJ/m^2^).
- In the case of the sample with the Nomex^®^ core, the impact strength on the laminate side (3.83 kJ/m^2^) and not much higher impact strength on the core side (5.40 kJ/m^2^).

In the case of the impact test, it was observed that for composites with Balsa and Rohacell^®^ cores, a higher impact strength was achieved when the impact occurred on the laminate side. In this scenario, the external CFRP cover layers effectively transmit and resist the tensile and compressive stresses generated by the impact. However, when the impact occurred on the core side, there was no layer specifically designed to carry the impact load, leading to progressive structural failure. In the case of the Rohacell^®^ core, significant energy dissipation occurred due to its closed-cell structure, which allows for energy absorption through cell wall deformation and localized crushing. This resulted in the CFRP/Rohacell^®^/CFRP structure achieving the highest impact strength. In the case of the Nomex^®^ composite, higher impact strength was observed when the impact occurred on the core side. This can be attributed to a slightly thicker cover layer resulting from the manufacturing process of this specific composite.

### 3.3. Determination of Flexural Strength

**Figure 15:**
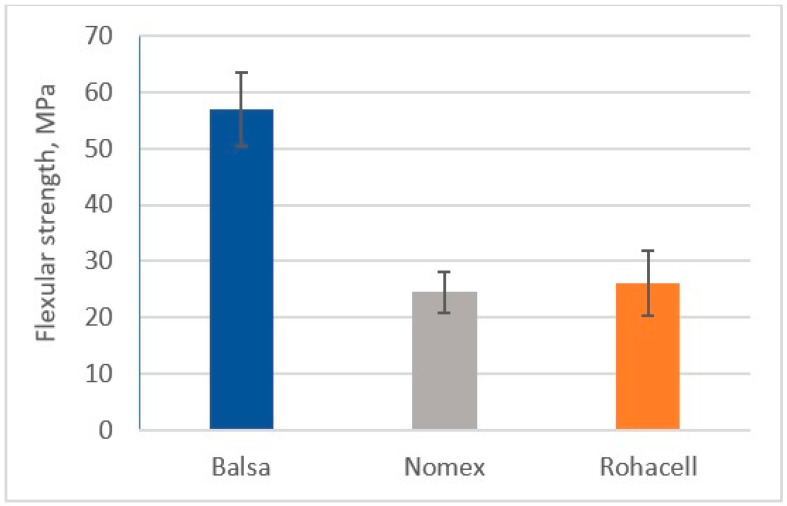
Flexular strength of sandwich composites.

**Figure 16:**
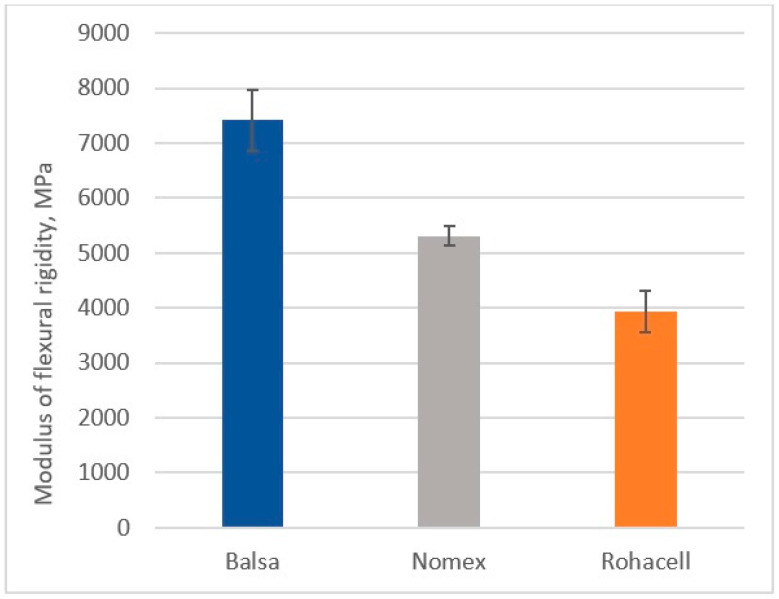
Modulus of flexular stiffness of sandwich composites.

Figure 15 and Figure 16 show the flexural strength results of the tested sandwich composites.

The Balsa-cored sandwich composite exhibited the highest flexural strength, reaching 56.9 MPa, while the Nomex^®^ and Rohacell^®^ core composites achieved strengths of 24.9 MPa and 26.2 MPa, respectively. The higher strength of the Balsa core can be attributed to its higher stiffness, which is directly related to the compactness of its structure. For the other cores, which have a more porous structure, their overall strength is more significantly influenced by the strength and stiffness of the cover material. With regard to the bending stiffness modulus, the CFRP/Balsa/CFRP system exhibited the highest value, around 7400 MPa. The composite with a Nomex^®^ core achieved a stiffness of around 5300 MPa, and the Rohacell^®^ core composite exhibited a stiffness of around 3900 MPa.

Similar studies to those conducted in this work were described by Ilvarasu et al. [27] and O. Castro et al. [28]. In both works, sandwich composites with Rohacell^®^ and Nomex^®^ cores were compared in bending tests. In the first work, laminates based on epoxy resin and glass fiber were used as cover laminates with two orientations of glass fabrics. The results obtained in bending tests were 24 MPa and 32 MPa, respectively, for composites with a Rohacell^®^ and Nomex^®^ core with a unidirectional fabric arrangement, and 41 MPa and 42 MPa for a glass fabric arrangement in a Bidirectional system [27]. The correlation of values corresponds to the results obtained in this research, i.e., 22 MPa and 23 MPa. O. Castro et al. [28] also conducted comparative studies of sandwich composites with different cores to compare their properties. Carbon fiber and epoxy resin-based laminates were used as face structures. In the case of bending test results, 62 MPa was obtained for the Rohacell^®^ core and 97 MPa for the Nomex^®^ core, respectively. Therefore, the observations from the comparative tests of the composites in the bending test also indicate better results when Nomex^®^ is chosen as the core, which is consistent with the results of the tests described in this manuscript. S. M. Zaharia et al. [30] conducted research on sandwich composites with a Balsa core, in which they performed a three-point bending test. The tests carried out on 10 samples with a thickness of 6 mm showed that the flexural strength ranges from 24 to 30 MPa. The stiffness modulus was obtained in the range of 3 to 6 GPa. Impact tests were also carried out, in which results in the range of 41 to 73 kJ/m^2^ were obtained. The results obtained in the bending test are about half lower than those obtained in this research, while the impact results are about 10 times higher, which may be due to a different proportion of the Balsa core to the laminate.

Better impact resistance results for the sandwich composite with a Rohacell core result from the cellular structure of the core, which is also a thin-walled structure. As a result of the sudden load from the hammer impact, the thin-walled cellular structure largely absorbs and dissipates the impact energy, which is converted into the work of deforming the cells of the Rohacell core structure. Studies conducted on three types of cores have shown that the core structure is quite important and, depending on the expected loads, a core should be used whose parameters and behavior have positive effects. In the case of the Balsa core, the layered structure of the wood and the significant stiffness are noticeable, which translates into good results in the QSPT test and in the bending test. In the case of the Rohacell core, which is a foam core but at the same time is a rigid structure, the composite using this core achieves weaker results in the QSPT test, but definitely better results in the impact test, where due to the porous structure, there is a significant dispersion of impact energy. The composite using the Nomex core achieves the weakest results in each of the tests carried out due to the low density of the honeycomb structure and its stiffness, which causes the composite with this structure to offer the least resistance to the indenter and poorly dissipates the impact energy.

## 4. Conclusions

Selected tests were conducted on the mechanical properties of the manufactured sandwich composites with three types of cores: Balsa, Rohacell^®^, and Nomex^®^. QSPTs were conducted to assess the energy absorption capacity, characterized by the area under the force-displacement curve, and the penetration resistance of the tested structures. In addition to the QSPT test, a flexural strength test and an impact test were carried out. Damage and failure characteristics of the composites were determined by puncture tests using a rounded end punch and an SPR parameter of five.

The CFRP/Balsa/CFRP sandwich composite exhibited the best strength parameters, with higher results in the bending test and QSPT than the composites with Rohacell^®^ and Nomex^®^ cores. While the CFRP/Balsa/CFRP arrangement exhibits the highest stiffness and strength due to its compact structure, it may have lower impact resistance compared to other core materials.

The sandwich composite with the CFRP/Rohacell^®^/CFRP system achieved the best results in the impact test, particularly when the load was applied from the direction of the cover laminate. In this test, this composite achieved values approximately two times better than the CFRP/Balsa/CFRP system, i.e., 11.9 kJ/m^2^ compared to 6.9 kJ/m^2^.

With regard to the CFRP/Nomex^®^/CFRP sandwich composite, this system exhibited a lower performance in the QSPT and impact tests compared to the other composites. However, it achieved a bending stiffness modulus that was approximately 20% higher than that of the system with a Rohacell^®^ core.

Based on performance tests of manufactured sandwich composites, the CFRP/Balsa/CFRP sandwich composite is recommended for aerospace components primarily subjected to bending and punching loads (e.g., structural panels and control surfaces) due to its superior strength and stiffness. However, its lower impact resistance should be considered and mitigated, if necessary, potentially through design modifications or localized reinforcement. For aerospace components requiring high impact resistance (e.g., leading edges and areas prone to debris impact), the CFRP/Rohacell^®^/CFRP sandwich composite is preferred due to its significantly higher impact resistance.

Future research should focus on enhancing the impact resistance of CFRP/Balsa/CFRP sandwich composites, given their excellent bending and punching performance. Potential avenues include exploring treatments or coatings for the Balsa wood, or investigating hybrid core structures combining Balsa with more impact-resistant materials. While Rohacell^®^ offers good impact resistance, its bending and QSPT performance is lower. Research should explore methods to improve these properties without compromising impact resistance, such as varying Rohacell^®^ foam density or structure, or developing hybrid cores. Combining Balsa and Rohacell^®^ into a hybrid core to achieve a synergistic effect warrants investigation. Furthermore, future research should investigate the long-term performance of these sandwich composites under relevant aerospace environmental conditions.

## Figures and Tables

**Table 1:** Physical properties of LH-289 resin [32].

Dynamic Viscosity	Density	Epoxy Index	Flash Point
500–900	1.12–1.16	0.51–0.56	>150
mPa·s, 25 °C	g/cm^3^	mol/1000	°C

**Table 2:** Comparison of densities of wood and similar materials [32].

Type of Wood	Density ρ, kg/m^3^
Balsa	40–180, aver. 110
Cork	210–350, aver. 280
Bamboo	300–400, aver. 350
Birchwood	540–780, aver. 660
Oak	700–1000, aver. 850

**Table 3:** Properties of Balsa, Rohacell^®^, and Nomex^®^ [36,37].

	Balsa	Rohacell^®^ 71 IG-F	Nomex^®^
Compressive strength [MPa]	12.8	1.50	2.4
Young’s modulus [MPa]	407	73	140
Shear strength [MPa]	2.98	1.3 *	1.2 **; 0.7 ***
Kirchhoff module	159	42	40 **; 25 ***

* (Plate), ** (longitudinal), and *** (transverse).

**Table 4:** The results of the quasi-static punching test of sandwich composites with three types of core.

		Type of Core	
	Balsa	Rohacell^®^ 71 IG-F	Nomex^®^
E_A_ [J]	12.74	8.09	5.42
F_max_ [N]	954	718	604
PSS [MPa]	2812	2116	1781
SEA [kJ/kg]	0.38	0.33	0.22

## Data Availability

The original contributions presented in this study are included in the article. Further inquiries can be directed to the corresponding author.

## Abbreviations

The following abbreviations are used in this manuscript:

QSPT	Quasi Static Puncture Test
CFRP	Carbon Fiber Reinforced Polymer
SPR	Support Span to Punch Diameter Ratio
PMI	Polymethacrylimide
PSS	Punch Shear Strength
SEA	Specific Energy Absorbed
IC	Impact Coefficient
